# Editorial: Insights in biosafety and biosecurity 2022/2023: novel developments, current challenges, and future perspectives

**DOI:** 10.3389/fbioe.2024.1380076

**Published:** 2024-03-12

**Authors:** Segaran P. Pillai, Stephen A. Morse

**Affiliations:** ^1^ Office of the Commissioner, Food and Drug Administration, Department of Health and Human Services, Silver Spring, MD, United States; ^2^ IHRC, Inc., Atlanta, GA, United States

**Keywords:** biosafety, biosecurity, novel developments, challenges, future perspectives

Biological research is an essential element for scientific advancements that underpin improvements in the quality and health of humans, agricultural animals and crops, domestic animals, and the environment. While biological research provides enormous benefits to society, there is a concomitant need for researchers to enhance their biosafety and biosecurity knowledge and practices as they continue to work with pathogens and toxins. Periodic assessment and reassessment of our biosafety and biosecurity framework and practices helps to ensure that they effectively address existing and emerging safety and security concerns while continuing to support scientific progress and innovation.

To address this need, in 2021, Frontiers developed a Research Topic entitled “*Insights in Biosafety and Biosecurity 2021: Novel Developments, Current Challenges, and Future Perspectives,”* which was co-edited by Pillai and Raybould (2023). Unfortunately, Dr. Raybould passed away on 5 October 2022 (Dritsas et al., 2023) and Dr. Morse was asked to co-edit the second volume of this Research Topic. The co-editors wish to acknowledge Dr. Raybould’s leadership and contributions to this important Research Topic.

For the second volume of this Research Topic, there were 16 submissions of which 9 were accepted and published (Policy and Practice Reviews, N = 4; Perspectives, N = 2; Original Research, N = 2; Hypothesis and Theory, N = 1). The authors of the published submissions were from multiple countries: Germany, United States, Poland, Netherlands, China, Singapore, Thailand, United Kingdom, Georgia, and Canada.

Engineering controls are one of the key measures to ensure the safety and health of the laboratorians. Kurth et al. discussed a previously unrecognized contradiction in the design of BSL-4 laboratories. For decades, it was suggested that both directional airflow and pressure differentials were essential safety measures to prevent the release of pathogens into the environment and to avoid cross-contamination between laboratory rooms. Despite the lack of an evidence-based risk analysis demonstrating increased safety by directional airflow and pressure differentials in BSL-4 laboratories, they were codified in various national regulations. The authors provided a detailed risk assessment by calculating pathogen mitigation in maximum contamination scenarios. Their results indicated that both directional airflow or a differential pressure gradient in airtight rooms within a secondary BSL-4 containment did not increase biosafety and were not necessary. Instead, they suggested that a reduction of pressure zones from the outside into secondary containment may provide sufficient environmental protection.

High-containment laboratories (HCLs) conduct critical research on high-consequence pathogens and provide diagnostic services for the diseases they cause. Modernization of HCLs has led to an increasingly cyber-connected laboratory infrastructure. Crawford et al. discussed the cybersecurity concerns specific to these HCLs to raise awareness among laboratory decision-makers and offer potential risk mitigation strategies.

Rutjes et al. observed that during the Covid-19 pandemic, the surge in demand for diagnostic tests had a substantial impact on biosafety and biosecurity. To prepare for the next pandemic, particularly in low- and middle-income countries, the authors have provided lessons learned, tools, and recommendations to improve biosafety and biosecurity practices to protect the front-line workers.

Ou and Guo provide an overview of current research on the application of synthetic biology in biomedicine and analyze the safety risks associated with this field. Based on their analysis, they propose fundamental principles for addressing these issues and offer practical recommendations for ethical governance, promoting the development and implementation of relevant policies, improving legal safeguards, and enhancing biocontainment.

Holub and Agena discussed biofoundries, which are highly automated facilities for processing biological specimens, and that have a major role in accelerating innovation and product development by bringing public and private stakeholders together to share resources and develop collaborations on national and international levels. The authors present an argument for expanding the scope for biofoundries to include roles in biosurveillance and biosecurity.

Sabra et al. analyzed the potential bioterrorism threat from *Bacillus anthracis* resulting from advances in synthetic biology, genome editing, information availability, and other emerging and enabling technologies. They concluded that rapid advances and availability of technologies has led to an ever-growing number and types of actors who could potentially weaponize *B. anthracis.*

Zimny proposed a reform of the European Union (EU) regulatory system for New Genomic Techniques (NGT) products to avoid placing EU researchers and investors at a disadvantage when compared to countries such as Argentina, Brazil, Canada, United Kingdom, and the United States.

Many countries have established and implemented regulations and policies for the accountability and control of high consequence pathogens and toxins that can have a significant impact on the economy as well as the health of agricultural animals and plants. In two contributions, (Pillai et al.; Pillai et al.). described a process using Multi-criteria Decision Analysis and Decision Support Framework logic tree approaches for evaluating: i) agricultural animal pathogens, and ii) plant pathogens to either support their inclusion on or exclusion from the list of agents for oversight and control. In contrast to lists of human pathogens where the impact on public health and safety were the primary factors for inclusion, non-biological criteria, i.e., economic consequences and impact on international trade agreements, were of paramount importance in these studies.

The need for enhanced biosafety and biosecurity practices continue to grow as we continue to discover new pathogens with high transmissibility, which can cause major outbreaks or the next epidemic or pandemic. This necessitates that scientists around the world conduct appropriate risk assessments and implement risk mitigation procedures to ensure the safety and health of the laboratorians, their family members, and the surrounding community. As we continue to enhance our biosafety and biosecurity practices, global support, collaboration, contribution, engagement and sharing of best practices are vital for success.

We would like to thank the authors for their contributions to this Research Topic on the importance of biosafety and biosecurity to ensure the safe, responsible, and secure conduct of biological science and research.
